# Oncoprotein 18 is necessary for malignant cell proliferation in bladder cancer cells and serves as a G3-specific non-invasive diagnostic marker candidate in urinary RNA

**DOI:** 10.1371/journal.pone.0229193

**Published:** 2020-07-02

**Authors:** Merle Hanke, Josephine Dubois, Ingo Kausch, Sonja Petkovic, Georg Sczakiel

**Affiliations:** 1 Institut für Molekulare Medizin, Universität zu Lübeck and UKSH, Lübeck, Germany; 2 Graduate School for Computing in Medicine & Life Sciences, Universität zu Lübeck, Lübeck, Germany; 3 Klinik für Urologie und Kinderurologie, Ammerland Klinik GmbH, Westerstede, Germany; Universitat des Saarlandes, GERMANY

## Abstract

**Background:**

Urine-based diagnostics indicated involvement of oncoprotein 18 (OP18) in bladder cancer. In cell culture models we investigated the role of OP18 for malignant cell growth.

**Methods:**

We analyzed 113 urine samples and investigated two human BCa cell lines as a dual model: RT-4 and ECV-304, which represented differentiated (G1) and poorly differentiated (G3) BCa. We designed specific siRNA for down-regulation of OP18 in both cell lines. Phenotypes were characterized by cell viability, proliferation, and expression of apoptosis-related genes. Besides, sensitivity to cisplatin treatment was evaluated.

**Results:**

Analysis of urine samples from patients with urothelial BCa revealed a significant correlation of the RNA-ratio OP18:uroplakin 1A with bladder cancer. High urinary ratios were mainly found in moderately to poorly differentiated tumors (grade G2-3) that were muscle invasive (stage T2-3), whereas samples from patients with more differentiated non-invasive BCa (G1) showed low OP18:UPK1A RNA ratios. Down-regulation of OP18 expression in ECV-304 shifted its phenotype towards G1 state. Further, OP18-directed siRNA induced apoptosis and increased chemo-sensitivity to cisplatin.

**Conclusions:**

This study provides conclusive experimental evidence for the link between OP18-derived RNA as a diagnostic marker for molecular staging of BCa in non-invasive urine-based diagnostics and the patho-mechanistic role of OP18 suggesting this gene as a therapeutic target.

## Introduction

Cancer of the urinary bladder, mainly a transitional cell carcinoma, is one of the most frequent human cancer types world-wide. Major challenges related to the treatment of bladder cancer (BCa) include a high recurrence rate of 50–80% [1] within a 5 years term after transurethral resection (TUR-B). Recurring tumors are often of an elevated grade and stage [2]. As a result, regular monitoring of this group of patients and prophylactic treatment appear to be necessary due to limited therapeutic options. In addition, the treatment of bladder cancer (BCa) depends on its stage. While non-muscle invasive forms of BCa can be removed by TUR-B of tumor tissue and its recurrence can be treated by immunotherapy with intra-vesicular delivery of attenuated *Bacillus Calmette–Guérin* (BCG) or intra-vesical chemotherapy, muscle-invasive forms of tumor demand more aggressive strategies. Chemotherapy includes platinum-based drugs like cis-diamminedichloridoplatinum (II), (henceforth referred to as cisplatin), as one of the standard chemotherapeutic agents for the treatment of metastatic BCa [3, 4]. Cisplatin effectively inhibits DNA synthesis by inducing DNA crosslinks [5, 6] and thus shows high toxicity. An active drug combination of cisplatin and the deoxynucleoside analog gemcitabine (2´,2´-difluorodesoxycytidine, dFdC) is particularly effective and considered as a suitable therapeutic option for the treatment of advanced BCa [7], especially metastatic disease.

In particular, the effectiveness of a variety of chemotherapy drugs, including cisplatin, is often substantially reduced because BCa tumors frequently develop a drug- or multiple drug-resistance (MDR) mechanism [8, 9]. Drug-resistant cells show, amongst other responses, an over-expression of anti-apoptotic genes [9], a sharp increase in the repair of damaged DNA [10], and an overexpression of enzymes involved in detoxification [11] and elimination of the drug [12]. Therefore, identifying new molecular targets and alternative classes of drugs, including oligonucleotide-based drugs [13, 14], is critical to improving survival in patients with advanced BCa. In addition to the need for alternative drugs, new types of diagnostics must be identified that enable earlier and ideally non-invasive detection of BCa. Further, there is a high level of clinical interest in objective and more accurate methods for tumor classification that can replace tissue-based histopathological staging.

Innovative diagnostic approaches are increasingly based on the non-invasive monitoring of BCa-specific tumor markers in urine. Promising markers for bladder cancer were based on RNA such as microRNAs and also sequences of cellular mRNAs [15–17]. Furthermore, we have shown that the analysis of the RNA composition in whole urine of BCa patients reveals specific and sensitive RNA-based tumor markers including ETS2 and uPA [18] as well as microRNAs [19].

Regarding OP18, also termed oncoprotein 18 and stathmin-1, immunohistochemical analyses of human donors and studies in the bladder cancer cell line T24 indicated that over-expression of OP18 is related to malignant cell characteristics. It is noteworthy that the role of increased expression of OP18 for tumor development and metastatic growth seems to be true also for other tumor types including esophageal squamous cell carcinoma [20, 21] and lung adenocarcinoma [22]. In summary, these studies warrant a closer look at OP18 transcripts as an RNA-based tumor marker in BCa.

In this study, we aimed to investigate whether differentially detectable RNAs in whole urine of BCa patients provide improved tumor markers *per se*. We also asked more specifically whether OP18-based RNA markers might indicate aberrant expression of OP18 and, hence, indicate malignant cells. For a deeper understanding of the tumor biology, we used two different BCa cell culture models to evaluate a possible involvement of OP18 gene expression in the tumorigenesis: The human cell lines ECV-304 [23–25] and RT-4 [26], representing well (G1) and poorly differentiated (G3) tumor states, respectively. To test whether model-based G1/G3-related phenotypes were in line with our results in liquid biopsies, we quantified RNA and protein levels of OP18 and performed several proliferation and cell viability assays under siRNA mediated suppression of OP18 mRNA. We also analyzed the influences of OP18 on apoptotic genes and chemo-sensitivity of G3 cells.

## Materials and methods

### Clinical samples and preparation of RNAs

This study was approved by the local ethical research committee in Lübeck, Germany (Ethikkommission der Universität zu Lübeck; chair, Prof. H. Raspe; scientific associate, Dr. A. Hüppe). All urine samples were obtained with written informed consent of the participants. The study was performed in accordance with the Declaration of Helsinki.

Tumor grading was determined by urinary bladder cystoscopy. In addition to biopsy, urine cytology was performed. All tumors identified were completely resected and classified pathologically according to the World Health Organization 1973 grading and staging system.

For investigation of urinary OP18 and uroplakin 1A (UPK1A) RNA levels, spontaneously voided urine of 113 donors was collected: 61 BCa patients (male:female, 3:1; G1 pTa, n = 13; G2+G3 pTa+pTis, n = 19; G2+G3 pT1, n = 12; G2+G3 pT2+pT3, n = 17; median age, 71 years), 37 healthy volunteers (male:female, 2:1; median age, 71 years), and 15 patients with infections of the urinary tract (male:female, 1:7; median age, 55 years). For details see S1 Table.

Spontaneously voided urine of donors was stabilized by immediately adding one volume of denaturing buffer (6.0 mol/l guanidinium isothiocyanate, 0.05 mol/l sodium acetate, and 0.5% N-lauroylsarcosine), freezing in liquid nitrogen, and storage at -80°C. Aliquots were withdrawn for RNA preparation as described recently [19]. Briefly, Total RNA from cells was prepared using the RNeasy Mini kit (Qiagen, Hilden, Germany). Urinary RNA was isolated using the RNeasy Midi Kit (Qiagen, Hilden, Germany). The procedure starts with an on-column hydrolysis of genomic DNA by treatment with DNase I. Prior to RNA isolation, urine samples were adjusted to pH 7.0 by 1 M HEPES buffer. Addition of a buffer termed ´RTL´ by the supplier (unknown composition) was replaced by adding recommended volumes of 70% ethanol and β-mercaptoethanol directly to urinary samples. RNA was eluted twice with 160 µl H_2_O and then lyophilized. Pellets were resolved in 20 µl H_2_O, and the quality of RNA was assessed by agarose gel electrophoresis (using 1 µg/ml ethidium bromide). Urinary RNA extracts (10 µl) or 400 ng total RNA from cells were used for cDNA synthesis and minus reverse transcriptase (non-RT) reaction.

### cDNA synthesis

Reverse transcription was performed in a total volume of 20 µl containing RNA extract (10 µl) and 300 ng of random hexamer primer (Invitrogen, Paisley, UK) according to the manufacturer’s instructions for SuperScript III™-driven reverse transcription (Invitrogen, Paisley, UK). The denaturation step was carried out at 75°C for a 10 min period. For non-RT control reactions, nuclease-free H_2_O was added instead of solutions of RNaseOut™ and reverse transcriptase.

It should be noted that RNA concentrations in urine are extremely low. For example, Nanodrop-based measurements of UV absorption cannot be used. However, the integrity of urine RNA seems to be good [27] and RT-qPCR-based detection is possible.

### Quantitative PCR (qPCR) and data analysis

Primers and TaqMan® probes were designed using Primer Express® software version 2.0 (Applied Biosystems, Darmstadt, Germany) or Primer3 software (Steve Rozen, Whitehead Institute for Biomedical Research, Cambridge, UK) and were purchased from Metabion (Martinsried, Germany) and Eurogentec (Seraing, Belgium), respectively. Primer sequences were checked for homology using the Blast software (www.ncbi.nlm.nih.gov/BLAST). Amplicon characteristics and software information are listed in the S2 Table.

All reactions were performed with the qPCR Core Kit (Eurogentec, Seraing, Belgium) in a total reaction volume of 10 µl in 384-well plates. A non-template control (nuclease-free H_2_O) was included for each amplicon to exclude contamination in every qPCR run. Each qPCR reaction was performed in triplicate. Quantitative PCR was carried out in a 7900HT thermal cycler (Applied Biosystems, Darmstadt, Germany): initial denaturation at 95°C for 10 min, followed by 50 cycles at 95°C for 15 sec and 60°C for 60 sec. PCR products were purified using the MinElute PCR Purification Kit (Qiagen, Hilden, Germany). Six serial 10-fold dilutions (10^1^–10^6^ copy numbers/reaction) were prepared in 10 mmol/l Tris/HCl (pH 8.0), 10 ng/ml polyinosinic acid potassium salt to generate standard curves. Data analysis was performed via the SDS 2.1 software (Applied Biosystems, Darmstadt, Germany) and the *threshold cycle (Ct)* values of amplified targets were transformed into absolute RNA copy numbers using the standard curves.

### Cell culture

The human urinary BCa cell line ECV-304 was cultivated in Medium 199 (with HEPES buffer + Earle's salts) containing 10% (vol/vol) fetal calf serum (FCS Gold). ECV-304 was originally established from an invasive, G3 BCa of an 82 years old Swedish female patient with a mutant p53 in 1970. It is a defined derivative of T24 [23–25] which we obtained from the DSMZ (´Deutsche Sammlung von Mikroorganismen und Zellkulturen´, Braunschweig, Germany), a repository for microorganism and cell lines. Cell identity was confirmed by DNA profiling by the DSMZ. RT-4 cells [26] were cultivated in RPMI 1640 medium supplemented with 10% (vol/vol) fetal calf serum. RT-4 cells were used as an *in vitro* model for differentiated G1 BCa. Both cell lines were cultivated without antibiotics at 37°C and 5% CO_2_ in a humidified incubator. All culture media and supplements were obtained from PAA Laboratories GmbH (Pasching, Austria). Control for Mycoplasma contamination was done using Venor®GeM Mycoplasma Detection Kit (Minerva Biolabs, Berlin Deutschland) according to the manufacturer’s instructions.

### Design and validation of siRNAs

Two small interfering RNAs targeting OP18 mRNA were designed in silico according to a systematic computational analysis of local target mRNA structures as described previously [28]. An extensive BLAST search indicated that both siRNA sequences were target-specific. Nucleotide sequences of the effective siRNA and a scrambled control siRNA without homology to human sequences are shown in the S3 Table. For the annealing of RNA strands (from IBA Goettingen, Germany), 20 µM of the sense and antisense strand, respectively, was denaturated in buffer (50 mmol/l potassium acetate, 1 mmol/l magnesium acetate, 15 mmol/l HEPES (pH 7.4)) at 90°C for 2 min followed by an annealing step at 37°C for 1 h. For transfection of siRNA, cells were seeded into tissue culture plates (12-well: 5 x 10^3^ ECV-304 cells or 1 x 10^4^ RT-4 cells; 96-well plates: 3 x 10^3^ ECV-304 cells). After 18–24 h, cells were transfected for 4 h at 37°C with 30 nM of siRNA using Lipofectamine 2000 in OPTI-MEM I according to the manufacturer’s instructions (Invitrogen, Paisley, UK).

### Phenotypic characterization and cell proliferation

Cell proliferation of untreated cells or cells post transfection with siRNA was measured in 12-well plates for 8 days or 96-well plates for 4 days. For this, ECV-304 cells or RT-4 cells were seeded in 12-well plates containing 1.0 ml of culture medium or in 96-well plates containing 0.1 ml of culture medium, respectively, and transfected as described above. Every 2 days, 500 µl for 12-well (and 50 µl for 96-well) plates of culture medium was replaced by fresh medium. At given time points, cells were harvested, and trypan blue-negative cells (0.4% trypan blue solution, Sigma Aldrich, Steinheim, Germany) were counted using a Neubauer hemocytometer. At day 1, 2, 3, and 4, cell viability was determined using a tetrazolium salt-based colorimetric assay (3-(4,5-dimethylthiazol-2-yl)-2,5-diphenyltetrazolium bromide (MTS). Cell culture medium was replaced by pH indicator-free culture medium containing 0.32 mg/ml MTS (Promega, Mannheim, Germany) and 0.0073 mg/ml phenazine methosulfate (Sigma-Aldrich). Cells were cultivated at 37°C for 2.5 h and A_490_ was determined by a Tecan Sunrise ELISA reader (Tecan Deutschland GmbH, Crailsheim, Germany).

Chemo-sensitivity of ECV-304 cells after siRNA-mediated suppression of OP18 was determined by treatment with cisplatin (Cisplatin-Teva®, Teva Pharma AG, Aesch, Switzerland) at 0, 1, 3, 6, 9, 12 µg/ml cisplatin over a period of 24 h starting at day 2 after transfection. Cell viability was determined using the MTS assay.

### Detection of apoptosis

ECV-304 cells were seeded (3 x 10^3^ cells, white-bottom 96-well plates) and transfected with siRNAs after 24 h as described above. Caspase 3/7 activity, was determined at day 3 and 4 post-transfection using the Caspase-Glo 3/7 Assay (Promega, Mannheim, Germany). The resulting fluorescence was used for normalization to cell viability. It was measured in a fluorometer (485_Ex_/527_Em_; Labsystems Fluoroscan Ascent, Helsinki, Finland). After addition of caspase 3/7 substrate and incubation at room temperature for 2.5 h, bioluminescence was detected in the fluorometer.

### Western blot analysis

ECV-304 cells were seeded in 12-well plates (5 x 10^4^ cells/well) and transfected after 24 h using the protocol described above. At 0, 1, 2, 3, and 4 days post-transfection, cells were harvested by trypsin treatment (0.05% trypsin/0.02% EDTA in 1x PBS for 5 min at 37°C), washed (PBS 1x ice-cold) and lysed (buffer containing 20% glycerol, 2% SDS, 125 mM Tris/HCl (pH 6.8), 5% beta-mercaptoethanol, and 0.02% (w/v) bromphenolblue). After denaturation (95°C for 5 min), samples were centrifuged (20 000 g for 1 min) and loaded onto a 16% SDS-polyacrylamide gel. Protein amounts of OP18 and beta-actin were quantified using a primary stathmin polyclonal antibody (1:1000; Cell Signaling Technology, NEB GmbH, Frankfurt/Main, Germany) and a polyclonal beta-actin antibody (1:1000; Abcam, Acris Antibodies, Hiddenhausen, Germany). Primary antibodies were detected by a secondary anti-rabbit IgG antibody conjugated with horseradish peroxidase (Dako, Glostrup, Denmark) and visualized via chemiluminescence (Pierce, Thermo Scientific, Karlsruhe, Germany).

### Statistical analysis

**Fig 1:**
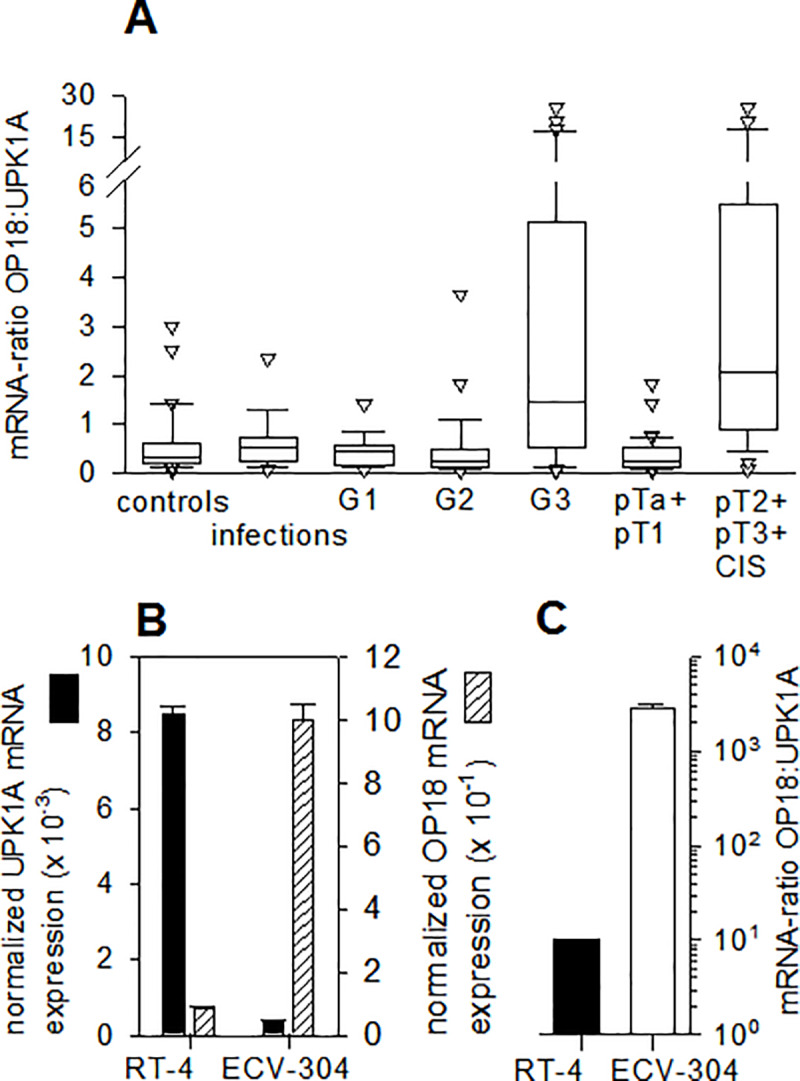
Urinary levels of the mRNA-based tumor marker OP18:UPK1A. (A), Box-Plot of the urinary mRNA ratio OP18:UPK1A. The qRT-PCR-based detection of OP18- and UPK1A mRNA was performed in triplicate. Donors were of different health status, including healthy individuals and individuals suffering from infections of the urinary tract or BCa as stratified according to grade (G1, G2 or G3) and stage, respectively (see Materials and Methods for details). Upper and lower limits of boxes and lines across boxes indicate the 75^th^ and 25^th^ percentiles and median, respectively. Error bars indicate the 90^th^ and 10^th^ percentiles. White triangles indicate outlying data points. The RNA ratio of OP18:UPK1A is significantly (*p* < 0.001) increased in patients with poorly differentiated (G3) BCa as well as invasive BCa (≥ pT2). (B), UPK1A- and OP18 mRNA expression in human BCa cell lines. Total RNA was prepared in triplicate from ECV-304 and RT-4 cells in the exponential phase of growth. Absolute mRNA copy numbers of OP18, UPK1A, and RPLP0 (60S acidic ribosomal protein P0), internal reference mRNA) were determined in triplicate by qRT-PCR using standard curves. Columns represent mean values ± standard deviation of UPK1A and OP18 mRNA copies after normalization to RPLP0 mRNA levels. (C), Ratio of OP18:UPK1A mRNA signals in RT-4 and ECV-304 cells. This ratio was calculated from single mRNA expression profiles presented in Fig 1B and shows a significant increase by approximately two orders of magnitude in poorly differentiated ECV-304 cells.

Depending on the size of patient groups two different non-parametric tests were applied: To compare OP18 to UPK1A signal ratios of three or more patient groups, the Kruskal-Wallis test (H-test) was used and for two different patient groups the unpaired Mann-Whitney-U test was used (Fig 1A). Statistical correlation of all other results was verified using student`s t-test. For all statistical tests, two-sided P values ≤ 0.05 were considered as statistically significant. Statistical analyses were performed using SPSS statistical software (Version 13.0, SPSS GmbH Software, Munich, Germany).

## Results

### Increased urinary OP18:UPK1A RNA-ratios are associated with invasive BCa

Total RNA was prepared from whole urine samples of healthy donors, patients with urinary tract infections, and patients with BCa. Analysis of urinary RNA revealed an RNA signal ratio OP18:UPK1A which is significantly (*p* < 0.001) increased in patients with poorly differentiated (G3) BCa as well as invasive BCa (≥ pT2) (Fig 1A).

While UPK1A-specific RNA sequences were less abundant in urine samples from G3 BCa patients when compared to G2 BCa patients, the high level of urinary OP18-specific RNA sequences increased with tumor invasiveness, thereby representing the determining factor for an increased OP18:UPK1A mRNA-ratio (Fig 1A).

Besides the potential suitability of OP18-derived RNA as a urinary marker for molecular staging, this observation indicated an involvement of OP18 gene expression in the tumorigenesis of BCa. To test whether model-based G1/G3-related RNA levels of OP18 and UPK1A were compatible with those in liquid biopsies, total RNA was prepared from both cell lines: The BCa-derived human cell lines RT-4 and ECV-304, representing well (G1) and poorly differentiated (G3) tumor states, respectively. Absolute copy numbers of OP18 and UPK1A as well as RPLP0 RNA (60S acidic ribosomal protein P0), serving as internal reference mRNA) were determined via qRT-PCR. This test revealed matching RNA levels of OP18 and UPK1A, respectively, and suggested the validity of this cell-based system to study the role of OP18 for malignant cell proliferation of bladder cancer (Fig 1B and 1C).

### Validation of siRNA tools against OP18 translation

**Fig 2:**
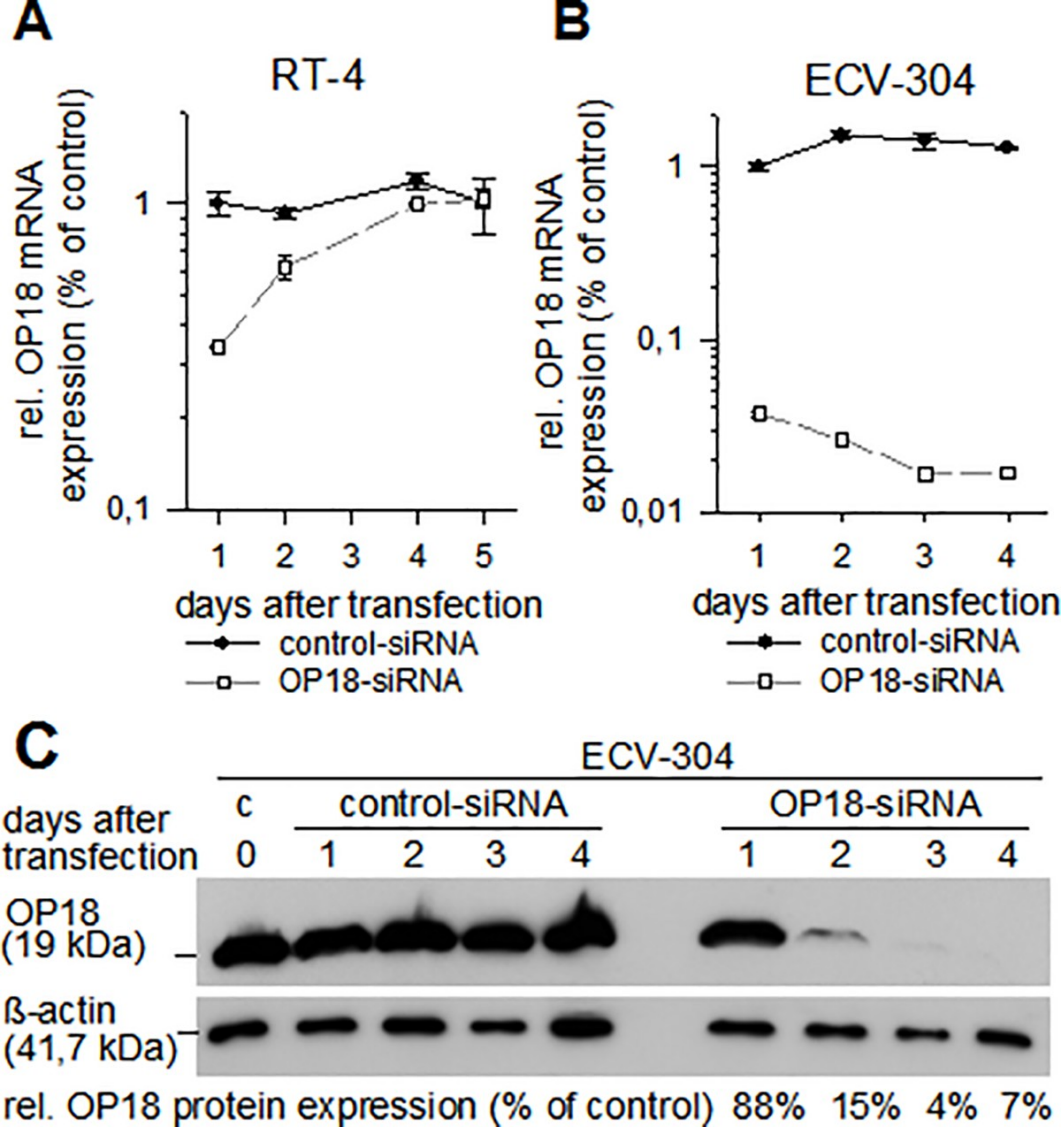
Validation of siRNAs. The two cell lines RT-4 (A) and ECV-304 (B) were transfected with OP18-directed siRNA or control siRNA at 30 nM. Total RNA was prepared after transfection at the indicated time points and levels of OP18- and RPLP0-mRNA were detected by qRT-PCR in triplicate. Symbols represent mean relative OP18 mRNA expression ± standard deviation as normalized to RPLP0. While RT-4 cells show transient and weak suppression of OP18 expression at days 1 and 2, ECV-304 cells show a substantial reduction of OP18 mRNA levels over a time period of 4 days which is statistically significant (see Materials & Methods). (C), relative OP18 protein amount in siRNA-treated ECV-304 cells. Cells were transfected with control-siRNA or OP18-siRNA (each at 30 nM). At 0, 1, 2, 3, and 4 days after transfection, cells were lysed, and OP18 protein and beta-actin were detected using western blotting. Signals of OP18 were normalized to beta-actin. Percentages of OP18 protein suppression in comparison to control cells is indicated in the lower panel of the blot.

The siRNA (sequences in S3 Table) was tested for suppression of OP18 expression in 5 x 10^4^ ECV-304 cells or 8 x 10^4^ RT-4 cells. The concentration dependency of siRNA-mediated suppression of OP18 at the transcriptional level showed an IC_50_ value of the most effective OP18-directed siRNA in ECV-304 cells below the lowest tested concentration of 3 nM [29]. Hence, in the following we used 30 nM of siRNAs in all experiments. Time-dependent siRNA-mediated inhibition of OP18 gene expression showed strong effects in the G3 model cell line ECV-304, but only moderate down-regulation of OP18 to levels of 31% at 24 h after transfection in RT-4 (Fig 2A).

Thereafter, the initial suppression of OP18 mRNA expression in RT-4 cells occurred to be transient as expected and disappeared within five days. This time course of suppression is observed in most transient tissue culture experiments in the use of siRNA. In contrast, in ECV-304 cells, the OP18 expression was inhibited efficiently to levels of approximately 2% of relative expression (Fig 2B). At the OP18 protein level, suppression was investigated for ECV-304 only (Fig 2C). A substantial decrease of OP18 protein signal was observed at days 2, 3, and 4 after transfection which relates to 15%, 4%, and 7% suppression, respectively (as compared to control siRNA).

### Suppression of OP18 is associated with reduced cell proliferation in ECV-304

**Fig 3:**
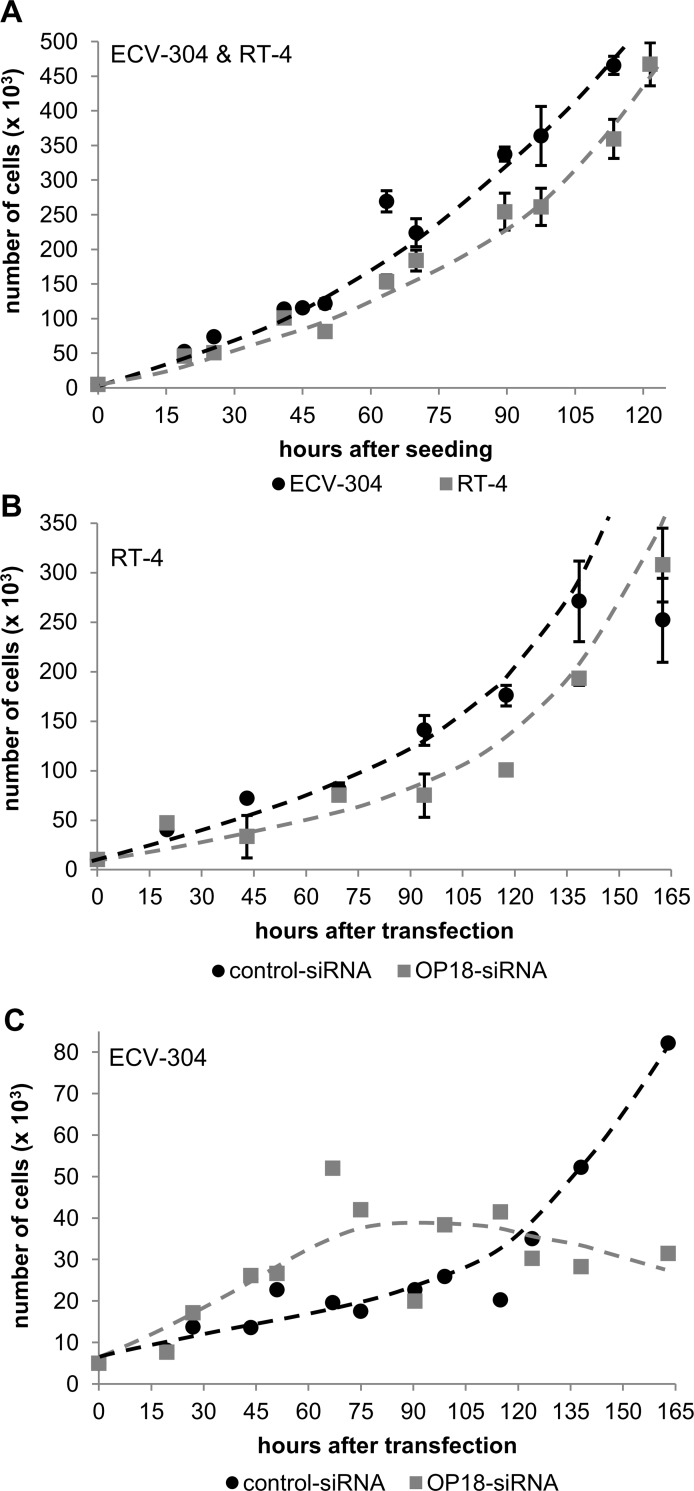
Growth curves of untreated or OP18-suppressed BCa cell lines RT-4 and ECV-304, respectively. (A), untreated RT-4 or ECV-304 cells, respectively. (B), siRNA-treated RT-4 cells and (C) siRNA-treated ECV-304 cells. Cells were seeded in triplicate (A), or transfected in triplicate with 30 nM of OP18-siRNA or control-siRNA (B, C). The number of viable cells at the indicated time points was determined by trypan blue staining. The data indicate mean values of 3 experiments ± standard deviation (A, B). For detection of proliferation rates of siRNA-treated ECV-304 cells (C), cells were counted in shorter intervals with one measurement by time point. Untreated RT-4 cells show slightly reduced cell growth compared to ECV-304 cells which is significant at and after 90 h after seeding (A). OP18-siRNA-treated RT-4 cells also display a minor decrease of doubling time compared to control-treated RT-4 cells (B). In contrast, ECV-304 cells show a major reduction of cell proliferation after transfection with OP18-directed siRNA versus control siRNA-treated ECV-304 cells with significant differences 130 h post transfection (C).

Next, we investigated a possible correlation of siRNA-mediated suppression of OP18 and cell proliferation in RT-4 and ECV-304 (Fig 3).

Analysis of cell growth of untreated and siRNA-treated RT-4 and ECV-304 cells was conducted. Untreated RT-4 cells showed slightly longer doubling time by a factor of approximately 1.5 compared to ECV-304 (Fig 3A). After transfection with functional or control siRNA, RT-4 cells still displayed nearly similar proliferation rates (Fig 3B). Conversely, proliferation of ECV-304 cells transfected with OP18-directed siRNA showed slightly enhanced cell growth initially followed by a complete stop of cell growth after day 5, when compared to treatment with control siRNA (Fig 3C). This time dependence of cell growth of siRNA-treated ECV-304 cells is noteworthy. However, we cannot explain the initially increased rates. They may be due to initial metabolic counter-reactions which may phenotypically increase functional OP18 protein levels when OP18 mRNA levels are suppressed while the absence of cell growth at later phases seems to indicate the importance of OP18 expression for cell proliferation.

### OP18-suppressed ECV-304 cells undergo apoptosis

**Fig 4:**
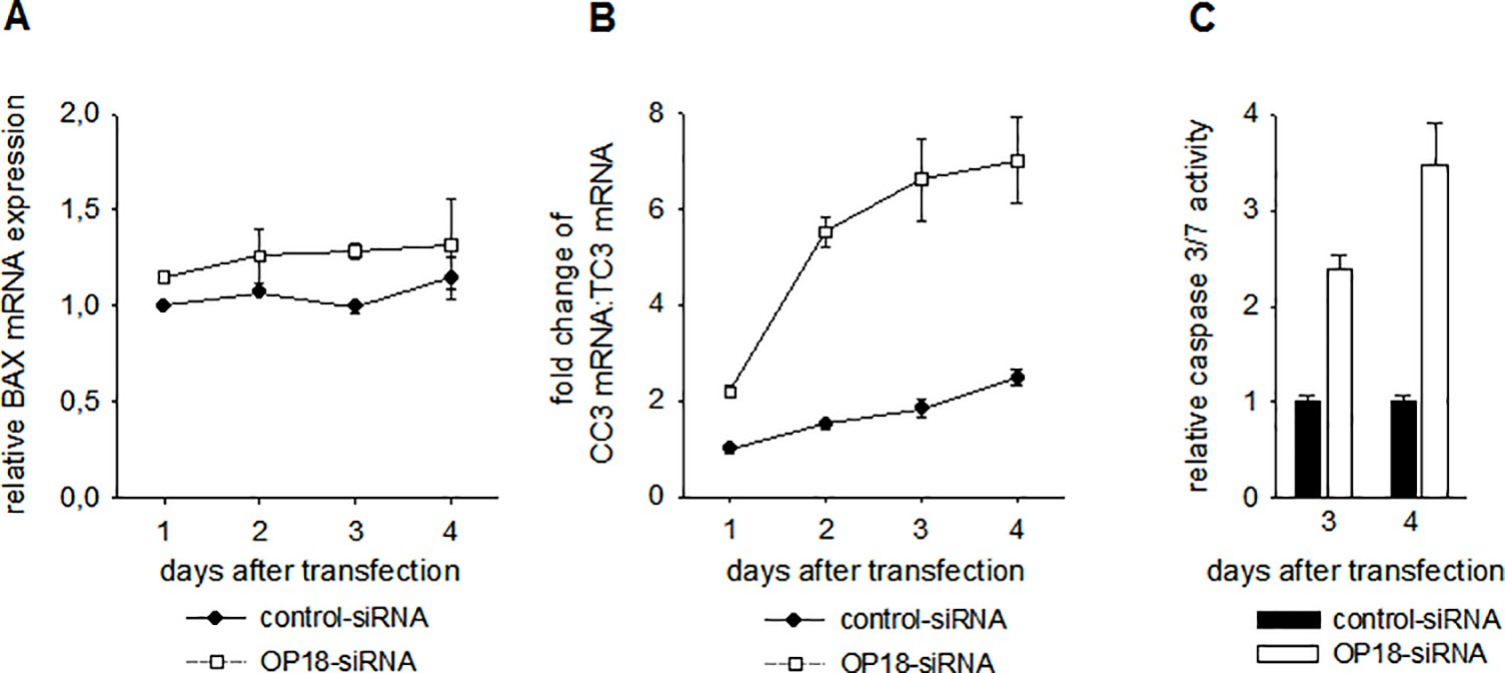
Apoptotic effects of OP18-suppression in ECV-304. Relative expression of the pro-apoptotic BAX mRNA (A) and increase (´fold change´) of the pro-apoptotic mRNA-ratio CC3:TC3 (B). ECV-304 cells were transfected in duplicate with 30 nM OP18- and control-siRNA and total RNA was prepared at 1, 2, 3, 4 days after transfection. Levels of BAX, CC3, TC3 and RPLP0 mRNA were detected in triplicate by qRT-PCR. Data indicate mean values ± standard deviation and are representative of three independent experiments. Levels of BAX mRNA of OP18-siRNA- or control-treated ECV-304 cells, respectively, differ marginally. Conversely, a significant difference was observed for the CC3:TC3 mRNA ratio on days 1 to 4. (C), Induction of caspase 3/7 activity after suppression of OP18. ECV-304 cells were transfected in triplicate with OP18- and control-siRNA (each 30 nM) and the relative caspase 3/7 activity was normalized to cell viability. The data indicate mean values ± standard deviation and are representative of three independent experiments. At day 3 and 4 after transfection, OP18-siRNA treated ECV-304 cells show a significant increase in caspase 3/7 activity compared to control cells.

To study the potential relationship between OP18 suppression and apoptosis, we analyzed the expression of apoptosis-related genes, (pro-apoptotic: BAX and CC3; anti-apoptotic: TC3) after transfection of ECV-304 cells with OP18-directed siRNA. While the expression level of BAX mRNA did not differ significantly between OP18-suppressed cells and controls (Fig 4A), the pro-apoptotic mRNA ratio CC3:TC3 increased progressively at day 4 after suppression of OP18 (Fig 4B).

To further investigate the induction of apoptosis in OP18-suppressed ECV-304 cells, relative caspase 3/7 activity was determined at protein level (Fig 4C). At day 3 and 4 after transfection, caspase 3/7 activity was enhanced by 2.5- and 3.5-fold, respectively.

### Suppression of OP18 increases chemo-sensitivity in ECV-304

**Fig 5:**
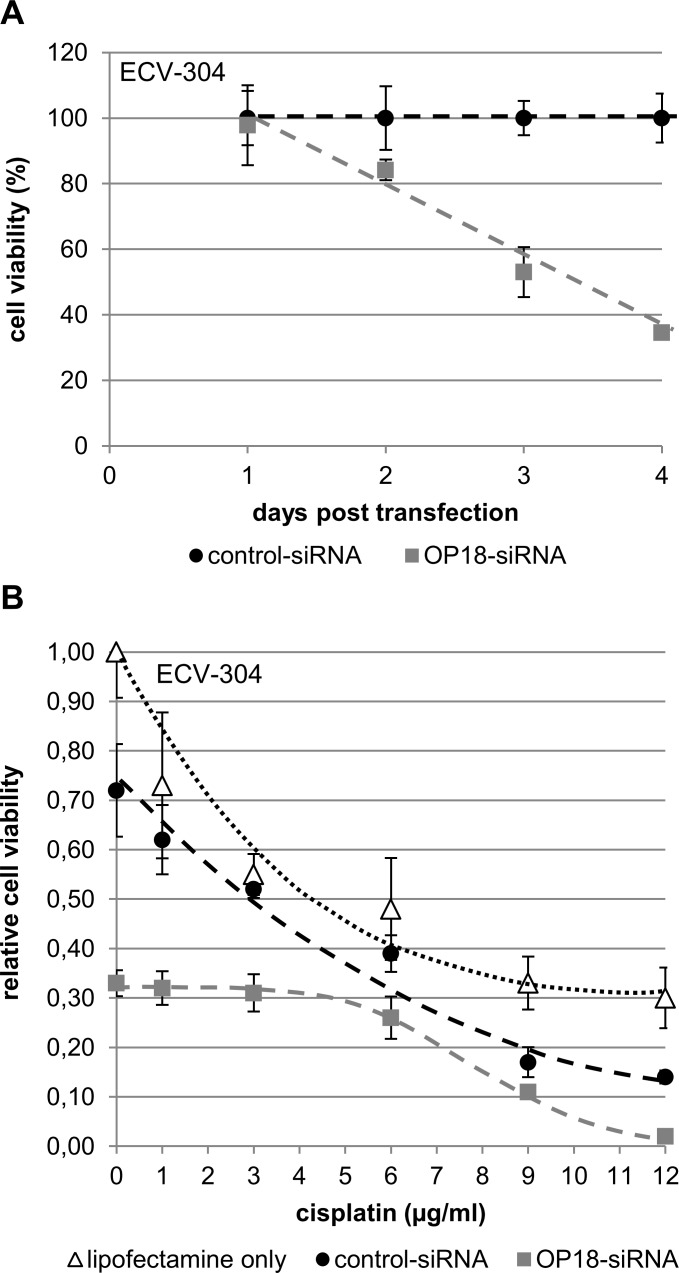
Chemo-sensitivity of BCa cells after suppression of OP18. ECV-304 cells were transfected in triplicate with each 30 nM of OP18- and control siRNA. (A), cell viability of siRNA-treated ECV-304 cells. The viability of ECV-304 cells was quantified with the colorimetric MTS-assay at 1, 2, 3, 4 days after transfection in triplicate (30 nM OP18- and control-siRNA). Symbols indicate mean values ± standard deviation of three independent experiments. Cell viability of OP18-siRNA treated ECV-304 was significantly decreased at days 2, 3, and 4 post transfection compared to control cells. (B) Cells were treated for 24 h with increasing concentrations of cisplatin (0, 1, 3, 6, 9, and 12 µg/ml) 48 h after transfection followed by quantification of cell viability. Data indicate mean values ± standard deviation and are representative of three independent experiments. ECV-304 cells treated with OP18-directed siRNA show a significant reduction of cell viability in the presence of cisplatin**,** particularly at highest cisplatin concentrations (12 µg/ml).

In a more therapeutically oriented perspective, the role of OP18 in sensitivity of ECV-304 for the chemotherapeutic agent cisplatin was studied (Fig 5).

Analysis of cell viability of OP18-suppressed ECV-304 cells showed a decrease of 35% at day 4 after transfection with functional siRNA when compared to cells transfected with control siRNA (Fig 5B).

Subsequently, ECV-304 cells were exposed to varying concentrations of cisplatin for 24h at day 2 post-transfection with siRNA. Notably, treatment with cisplatin had an additive effect on loss in relative viability in ECV-304: At high concentrations of cisplatin (9 and 12 µg/ml), a progressively severe effect was observed on cell viability of OP18-suppressed cells as compared to controls. At a cisplatin concentration of 12 µg/ml, the decrease in cell viability of OP18-suppressed ECV-304 cells was in the order of one magnitude while in the absence of cisplatin, this was only approximately twofold (Fig 5A, and 5B, “0” µg/ml cisplatin). At low concentrations of cisplatin (1, 3 and 6 µg/ml), the effect on relative cell viability was similar in OP18-suppressed cells and controls.

## Discussion

### Functional insights into the biological role of OP18 and its involvement in malignant cell proliferation

In this study we aim to shed light on the role of OP18 for aberrant cell proliferation and as a diagnostic marker for BCa. On the cell culture side, the dual cell model, ECV-304 cells and RT-4 cells, respectively, suggests that OP18 acts as a crucial determinant of malignant cell growth. Major evidence is derived from siRNA-mediated suppression of OP18 gene expression at the mRNA and at the protein level in ECV-304 cells which represent a poorly differentiated (G3) BCa state. OP18 suppression decreased the fast proliferation of ECV-304 cells and resulted in increased apoptosis. Notably, we observed an increased chemo-sensitivity when OP18 suppressed cells were treated with cisplatin which extends the knowledge about the tumor biology of OP18 and its role as a therapeutic target. On the mechanistic level, these tumor-related characteristics of OP18 in BCa-derived tissue culture cells are consistent with reports on the oncogenic properties of OP18 in other tumor types including hepatocellular carcinoma [30], lung adenocarcinoma [22], and prostate cancer [31]. OP18 is a cytosolic phosphoprotein which plays a key part in the regulation of cell proliferation [32, 33]. During anaphase of mitosis, OP18 depolymerizes microtubules and prevents the polymerization of tubulin heterodimers. It was originally identified in leukemia, where OP18 was found to be highly expressed [34, 35]. In the past, OP18 has been studied most extensively in breast, gastric and lung cancer due to the high prevalence of these diseases [36–42].

Prognostic value has been assigned to OP18 in different tumor entities based on tissue biopsies [43–46]. Recently, Hemdan *et al*. investigated the role of OP18 in BCa [47] by using a commercial set of OP18-directed siRNAs, not overlapping with the siRNAs used in this study and reported findings which are consistent with this study. However, in contrast to Hemdan *et al*., we studied urine biopsies and two cell culture models by molecular methodology rather than tissues and histopathological staining. The use of whole urine samples is advantageous because urine is readily available and can be collected, delivered, and stored simply. BCa-specific markers can be detected in a standardized manner by RT-qPCR [48], whereas histopathological examinations of tumor tissue strongly depend on pathological expertise and software for pattern recognition and image analysis [49, 50].

### OP18 as a potential therapeutic target in bladder cancer

In this study, the suppression of OP18 gene expression by siRNA was observed on the levels of mRNA and protein and this seems to be related to decreased cell proliferation indicating a therapeutic potential. The phenotypic gene function analysis of OP18 described here suggests to further test a wider repertoire of OP18-directed inhibitors to therapeutically address more advanced tumor stages of bladder cancer and their conversion to less malignant stages. We anticipate that instillation of drugs into the bladder produces a scenario of drug application that is closer to a local rather than a systemic application which implies several fundamental advantages such as increased local concentration of the drug and, hence, increased delivery to tumor cells, higher stability, and decreased side effects.

### OP18 as tumor marker for urinary bladder cancer

The findings described in this study suggest OP18 as a molecular marker for BCa and as a molecular contributor to the disease. We assume that OP18-specific RNA contained at elevated levels in urine of BCa patients at advanced tumor stages reflects increased OP18 expression in tumor tissue. It is reasonable to assume that RNA sequences of OP18 in urine serve as a tumor marker that directly monitors the disease. In order to improve sensitivity and specificity of this marker, combinations of RNA-based markers might give rise to improved diagnostics of BCa and in a non-invasive setting, i.e. based on urine samples. It has to be noted that RNA concentrations in urine are too low to be measured by conventional quantitative methods. This is one of the major reasons to define ratios of markers which are independent of concentrations. For example, the analysis of urinary mRNAs in this study revealed an improved relationship between the mRNA-ratio OP18/UPK1A and poorly differentiated (G3) and muscle-invasive (≥ pT2) BCa cancer states. Our data strongly indicate that this strategy has great potential for future accurate and non-invasive diagnostic developments in case of bladder cancer and beyond.

## Conclusions

OP18 expression seems to be necessary for malignant cell proliferation in human cells derived from bladder cancer. In a diagnostic perspective, urine RNA levels (OP18:UPK1A) serve as a molecular marker for the invasive disease. In mechanistic terms, over-expression of OP18 seems to be necessary for maintaining the malignant state of BCa cells as its suppression results in an increased chemo-sensitivity and apoptosis. Hence, the gene expression of OP18 represents a rational therapeutic target and diagnostic readout.

## Supporting information

S1 Table Clinical characteristics of patients and healthy donors. (DOCX)

S2 Table Sequences and characteristics of qPCR amplicons. (DOCX)

S3 Table Most effective siRNA sequence and scrambled RNA sequence. (DOCX)

S1 Raw images (PDF)
