# Supplementary material for: Oncoprotein 18 is necessary for malignant cell proliferation in bladder cancer cells and serves as a G3-specific non-invasive diagnostic marker candidate in urinary RNA
Source: PLoS One. 2020 Jul 2;15(7):e0229193. doi: 10.1371/journal.pone.0229193 (PMC7332083; doi:10.1371/journal.pone.0229193)
Supplement: S1 Table — (DOCX) [file pone.0229193.s001.docx]

**S1 Table. Clinical characteristics of patients and healthy donors.**

| ***Patients with urinary bladder cancer*** | |  |  |
| --- | --- | --- | --- |
| *Age range (median)* | *% of population* | *Male (n)* | *Female (n)* |
|  |  |  |  |
| 40-60 (57) | 24.6 | 11 | 4 |
| 61-70 (69) | 21.3 | 10 | 3 |
| 71-80 (74) | 34.4 | 18 | 3 |
| 81-90 (84) | 16.4 | 4 | 6 |
| <90 (95) | 3.3 | 1 | 1 |
|  |  |  |  |
| ***Healthy donors*** | |  |  |
| *Age range (median)* | *% of population* | *Male (n)* | *Female (n)* |
|  |  |  |  |
| 40-60 (53) | 16.2 | 4 | 2 |
| 61-70 (65) | 51.4 | 11 | 8 |
| 71-80 (76) | 16.2 | 6 | / |
| 81-90 (82) | 16.2 | 2 | 4 |
|  |  |  |  |
| ***Patients with urinary tract infections*** | |  |  |
| *Age range (median)* | *% of population* | *Male (n)* | *Female (n)* |
|  |  |  |  |
| 20-40 (39) | 20.0 | 1 | 2 |
| 41-60 (46) | 13.3 | / | 2 |
| 61-70 (64) | 33.3 | / | 5 |
| 71-90 (79) | 33.3 | 1 | 4 |
|  |  |  |  |
