# Supplementary material for: Oncoprotein 18 is necessary for malignant cell proliferation in bladder cancer cells and serves as a G3-specific non-invasive diagnostic marker candidate in urinary RNA
Source: PLoS One. 2020 Jul 2;15(7):e0229193. doi: 10.1371/journal.pone.0229193 (PMC7332083; doi:10.1371/journal.pone.0229193)
Supplement: S2 Table — (DOCX) [file pone.0229193.s002.docx]

**S2 Table. Sequences and characteristics of qPCR amplicons.**

| **Target mRNA** | **Accession No.** | **Sequence: 5’*→* 3’**   1. **forward primer** 2. **reverse primer** 3. **TaqMan® probe^1^** | **Amplicon**  **length (bp)** | **E^B^** | **R^2^ of standard curve^C^** |
| --- | --- | --- | --- | --- | --- |
| BAX |  | 1. TGGAGCTGCAGAGGATGATT |  |  |  |
|  | NM_138761 | 2. AGCTGCCACTCGGAAAAAGA | 71 | 1.98 | 0,9994 |
|  |  | 3. **FAM**-CCGCCGTGGACACAGACTCCC-**BHQ-1** |  |  |  |
|  |  |  |  |  |  |
|  |  | GTCTGCAGAGCTGGCAAAAG |  |  |  |
| CC3 | NM_006410 | TCAACCTTGGCTTCTACTTCTCC | 123 | 1.98 | 0.9996 |
|  |  | **YY**-TGGAGGGTGCAAACATTTCAACTTGC-**BHQ-1** |  |  |  |
|  |  |  |  |  |  |
|  |  | CTGGAGAAACTGCTGCCTCAT |  |  |  |
| RPLP0 | NM_001002 | CACCTTATTGGCCAGCAACA | 99 | 1.98 | 0.9988 |
|  |  | **FAM**-CCGGGGGAATGTGGGCTTTG-**BHQ-1** |  |  |  |
|  |  |  |  |  |  |
|  |  | AAAGACGCAAGTCCCATGAAG |  |  |  |
| OP18 | NM_203401 | AGCTTCCATTTTGTGGGTCAG | 146 | 1.95 | 0.9992 |
|  |  | **FAM**-GCAGCTGGCTGAGAAACGAGAGCA-**BHQ-1** |  |  |  |
|  |  |  |  |  |  |
|  |  | GGAAGCTCACCTTCGACGAG |  |  |  |
| TC3 | AF092095 | AGCATTGCAACAGGCACAAA | 242 | 1.95 | 0.9988 |
|  |  | **FAM**-CAGAGGGAAAGCTGGGGCGG **BHQ-1** |  |  |  |
|  |  |  |  |  |  |
|  |  | CGTCATGATTGAGCAAGAATGC |  |  |  |
| UPK1A | NM_007000 | CGGAAGGCTGACGTGAAGT | 72 | 1.95 | 0.9992 |
|  |  | **FAM**-TGGCACATCTGGTCCCATGGA-**BHQ-1** |  |  |  |

^1^FAM = 6-Carboxyfluorescein; BHQ = Black Hole Quencher™; YY = Yakima Yellow
