# Supplementary material for: Oncoprotein 18 is necessary for malignant cell proliferation in bladder cancer cells and serves as a G3-specific non-invasive diagnostic marker candidate in urinary RNA
Source: PLoS One. 2020 Jul 2;15(7):e0229193. doi: 10.1371/journal.pone.0229193 (PMC7332083; doi:10.1371/journal.pone.0229193)
Supplement: S3 Table — (DOCX) [file pone.0229193.s003.docx]

S3 Table. Most effective siRNA sequence and scrambled RNA sequence.

|  | siRNA | sequence 5´*→* 3´ |
| --- | --- | --- |
| OP18-siRNA | Sense | ACG AGA CUG AAG CUG ACU A dtdt* |
|  | antisense | UAG UCA GCU UCA GUC UCG U dtdt* |
| Control-siRNA | scrambled sense | CGG ACG CAC UGG UCU GAC CGG dtdt* |
|  | scrambled antisense | CCG GUC AGA CCA GUG CGU CCG dtdt* |

^*^ 2´deoxyribothymidines which were used instead of uridines; overhangs are underlined
