# Supplementary figures and images for: Oncoprotein 18 is necessary for malignant cell proliferation in bladder cancer cells and serves as a G3-specific non-invasive diagnostic marker candidate in urinary RNA
Source: PLoS One. 2020 Jul 2;15(7):e0229193. doi: 10.1371/journal.pone.0229193 (PMC7332083; doi:10.1371/journal.pone.0229193)

| ECV-304                 |   |               |   |   |   |   |            |   |   |   |  |
|-------------------------|---|---------------|---|---|---|---|------------|---|---|---|--|
| days after transfection | c | control-siRNA |   |   |   | M | OP18-siRNA |   |   |   |  |
|                         | 0 | 1             | 2 | 3 | 4 |   | 1          | 2 | 3 | 4 |  |

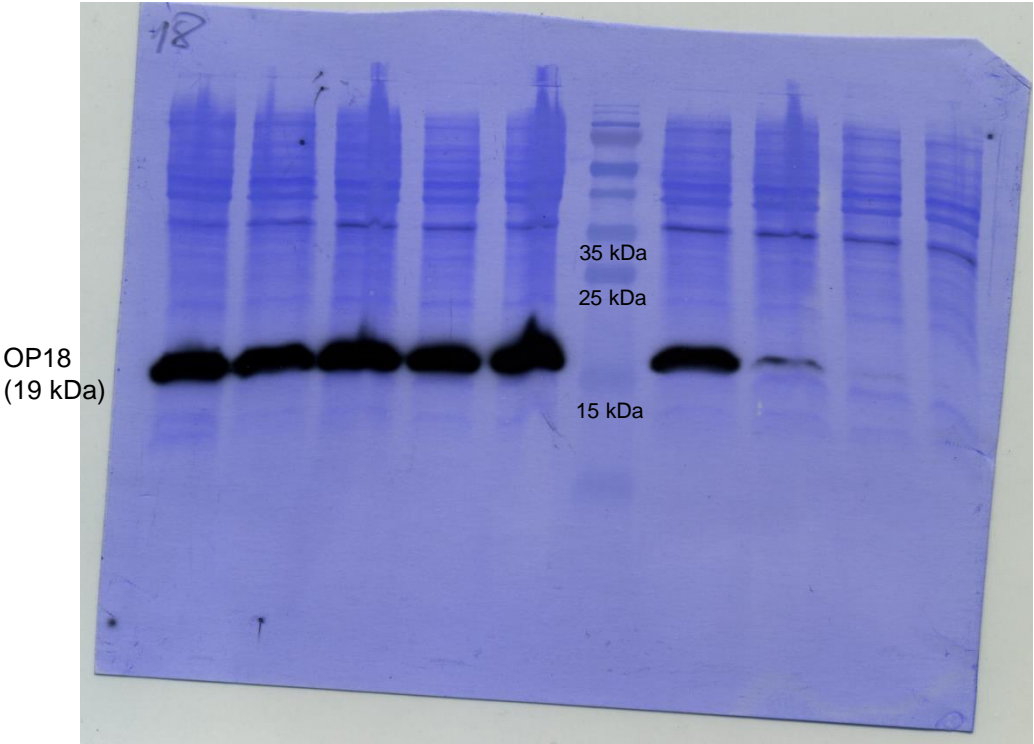

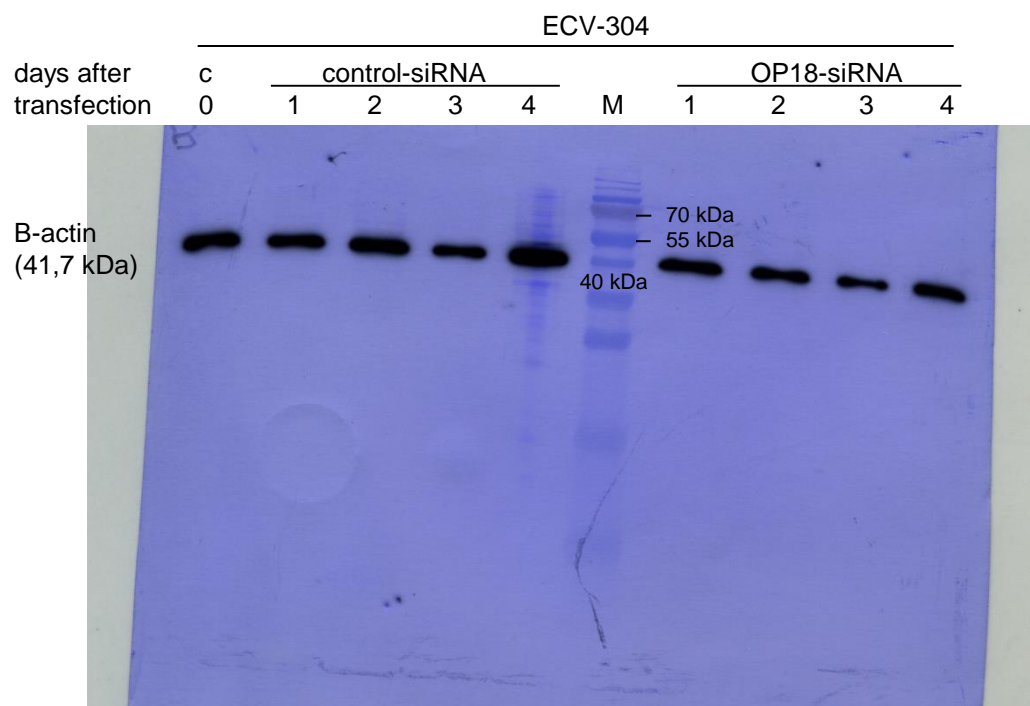

Supplement: S1 Raw images — (PDF) [file pone.0229193.s004.pdf]
